# Evaluation of the Biological Properties of an Optimized Extract of *Polygonum cuspidatum* Using Ultrasonic-Assisted Extraction

**DOI:** 10.3390/molecules28104079

**Published:** 2023-05-13

**Authors:** Gabriela Fletes-Vargas, Rogelio Rodríguez-Rodríguez, Neith Pacheco, Alejandro Pérez-Larios, Hugo Espinosa-Andrews

**Affiliations:** 1 Laboratorio de Nanomateriales, Agua y Energía, Departamento de Ingenierías, Centro Universitario de Los Altos, Universidad de Guadalajara, Tepatitlán de Morelos 47600, Mexico; ana.fletes3623@alumnos.udg.mx (G.F.-V.); alarios@cualtos.udg.mx (A.P.-L.); 2 Unidad de Tecnología Alimentaria, Centro de Investigación y Asistencia en Tecnología y Diseño del Estado de Jalisco, Zapopan 45019, Mexico; 3 Departamento de Ciencias Naturales y Exactas, Centro Universitario de los Valles (CUVALLES), Universidad de Guadalajara, Ameca 46600, Mexico; 4 Centro de Investigación y Asistencia en Tecnología y Diseño del Estado de Jalisco CIATEJ, A.C. Subsede Sureste, Parque Científico Tecnológico de Yucatán, Mérida 97302, Mexico; npacheco@ciatej.mx

**Keywords:** antioxidant capacity, resveratrol, *Polygonum cuspidatum*, ultrasonic-assisted extraction, response surface methodology, infusion

## Abstract

Phytochemicals are natural compounds found in plants that have potential health benefits such as antioxidants, anti-inflammatory and anti-cancer properties, and immune reinforcement. *Polygonum cuspidatum* Sieb. et Zucc. is a source rich in resveratrol, traditionally consumed as an infusion. In this study, *P. cuspidatum* root extraction conditions were optimized to increase antioxidant capacity (DPPH, ABTS+), extraction yield, resveratrol concentration, and total polyphenolic compounds (TPC) via ultrasonic-assisted extraction using a Box–Behnken design (BBD). The biological activities of the optimized extract and the infusion were compared. The optimized extract was obtained using a solvent/root powder ratio of 4, 60% ethanol concentration, and 60% ultrasonic power. The optimized extract showed higher biological activities than the infusion. The optimized extract contained 16.6 mg mL^−1^ resveratrol, high antioxidant activities (135.1 µg TE mL^−1^ for DPPH, and 230.4 µg TE mL^−1^ for ABTS+), TPC (33.2 mg GAE mL^−1^), and extraction yield of 12.4%. The EC_50_ value (effective concentration 50) of the optimized extract was 0.194 µg mL^−1^, which revealed high cytotoxic activity against the Caco-2 cell line. The optimized extract could be used to develop functional beverages with high antioxidant capacity, antioxidants for edible oils, functional foods, and cosmetics.

## 1. Introduction

Nutraceutical is a term derived from “nutrition” and “pharmaceutics”, used for compounds isolated from herbal products with biological activity. These compounds provide health benefits, especially for preventing and treating diseases such as cancer, diabetes, cardiovascular and neurological disorders [1,2]. These diseases are associated with the generation and accumulation of reactive oxygen species (ROS) produced by cellular oxidative stress [3,4]. Antioxidant compounds can inhibit or decrease oxidation processes that affect biomolecules such as proteins, lipids, and DNA [5]. Antioxidants can protect cells against oxidation by blocking the initiation phase of radical production or neutralizing radicals. Commonly, herbal plants contain antioxidant properties due to the presence of bioactive compounds. *Polygonum cuspidatum Siebold* & *Zucc.* belongs to the Polygonaceae family and grows widely in Asia and North America. It has been used for centuries in China and Japan as an herbal medicine to treat inflammatory diseases, hepatitis, tumors, diarrhea, arthralgia, chronic bronchitis, amenorrhea, hypertension, neuroprotector, and hypercholesterolemia. In addition, its ethanolic extracts have estrogenic and antiviral activities against hepatitis B viruses and SARS-CoV-2 omicron [6,7]. The root of *P. cuspidatum* contains many secondary metabolites with biological efficacy. These compounds have been identified as stilbenes, including resveratrol, piceid, and emodin.

Resveratrol is one of the most highly investigated antioxidant molecules [8]. Resveratrol (3,5,4′-trihydroxy-stilbene) is a polyphenolic molecule found in many foods such as grapes, mulberries, peanuts, cereals, vegetables, flowers, and roots [9,10]. Resveratrol is a secondary metabolite that confers protection against pathogenic attack, UV radiation and environmental stress, heavy metals, and in some cases, climate change [11]. Resveratrol reduces the formation of intracellular ROS and oxidative damage, thereby providing several biological activities: anti-inflammatory, antioxidant, anti-aging, anti-tumor, and anti-mutagenic [12]. However, the therapeutic potential and bioavailability of resveratrol are limited due to its low water solubility [13]. Piceid is a stilbenoid with a neurological protection effect that has been reported at concentrations 10 or 15 times higher than resveratrol [14]. In addition, its bioavailability is lower compared to resveratrol due to intestinal cells absorbing piceid slowly, and this process requires glycosidases [15]. Emodin is an anthraquinone located in the rhizome, and quercetin is a flavonoid in leaves and stems. Polyphenols are commonly found in flowers [16,17].

Traditionally, all vegetable parts of *P. cuspidatum* are consumed as tea beverages or infusions with medicinal aims. However, the extraction of phenolic compounds from *P. cuspidatum* can be performed using Soxhlet extraction, which has the disadvantage that the extraction is performed at elevated temperatures for a long time, which can degrade the polyphenols [18]. In addition, polyphenol extraction can use organic solvents such as acetone, ethanol, methanol, and ethyl acetate; however, organic solvents may not be efficient [19]. Recently, ultrasound-assisted extraction technology (UAE) has been employed to increase extraction yields and, in some cases, to perform more selective extractions. UAE is an innovative extraction technique in which the sample can be mixed with organic solvents at a controlled temperature, reducing the extraction time [20]. The release of phytochemicals is due to the rupture of the cell walls by ultrasound waves, a phenomenon called cavitation. Extraction of polyphenols with UAE is higher in yield (6–35%) and more time-saving than other traditional techniques [21,22].

Response surface methodology (RSM) is a mathematical and statistical technique widely used to investigate multiple parameters and their possible interactions to optimize processes [23]. RSM reduces the number of experimental runs and the time required to investigate the optimal conditions for extraction [24]. Kuo et al. [18] optimized the extraction conditions of phenolic compounds from *P. cuspidatum* using multiple RSM. The authors reported that temperature and ethanol concentration impacted the extraction yields of the bioactive compounds. In addition, using supercritical carbon dioxide technology, Ruan et al. [25] reported that *P. cuspidatum* extracts showed high scavenging capacity. However, there are few reports about the biological activities of ethanolic extracts of *P. cuspidatum*.

This study aimed to optimize *P. cuspidatum* root extraction conditions to increase antioxidant capacity (DPPH, ABTS+), extraction yield, resveratrol concentration, and total polyphenolic compound content via UAE using a Box–Behnken design (BBD). The independent variables were solvent/root powder ratio, ethanol concentration, and ultrasonic power. We hypothesized that the interaction of the independent variables would increase the antioxidant capacity of the extract, extraction yield, polyphenolic compound content, and resveratrol concentration compared to the infusion. Thus, the biological activities of the optimized extract and the traditional infusion were compared.

## 2. Results

### 2.1. Model Fitting from Extracts of P. cuspidatum Root

Table 1 shows the experimental values of antioxidant capacity (DPPH and ABTS+), TPC, resveratrol concentration, and extraction yield obtained by UAE from the interaction variables: solvent/root-powder ratio (*X*_1_), ethanol concentration (*X*_2_), and ultrasonic power (*X*_3_). According to the experimental values, the scavenging capacities for DPPH and ABTS+ ranged from 51.1 to 135.1 µg TE mL^−1^ and 119.5 to 230.4 µg TE mL^−1^, respectively. The data obtained for total polyphenolic compounds ranged from 5.8 to 33.2 mg GAE mL^−1^. The resveratrol content in crude extract and the extraction yield ranged from 12.0 to 16.7 mg mL^−1^ and 2.74% to 12.43%, respectively. A reduced quadratic polynomial was used to predict the experimental data, shown in Equation (1).

Table 2 shows the ANOVA results for the responses and interactions of the independent variables. Regarding antioxidant activities (ABTS^+^ and DPPH), variables *X*_1_ and *X*_2_*^2^* showed negative effects, while variable *X*_2_ had highly significant positive effects (*p* < 0.001). Meanwhile, variable *X*_3_ did not show a significant effect; however, it contributed positively to the antioxidant activities. The analysis indicated that the determinant coefficients (R^2^) were 0.9612 for ABTS^+^ and 0.9593 for DPPH. The regression model explained 96.12% and 95.93% of the responses for ABTS^+^ and DPPH, respectively (*p* < 0.05). The TPC showed a linear impact for variables *X*_1_ and *X*_2_, with an R^2^ = 0.8581_,_ able to explain 85.81% of the fitted regression model (*p* < 0.05). Ethanol concentration was the most critical parameter for increasing the resveratrol content in the extract. The lineal variable *X*_2_ showed a highly significant positive effect (*p* < 0.001), while variable *X*_2_^2^ showed a highly significant negative effect (*p* < 0.001). According to the F-value, the impact of the linear *X*_2_ is higher than the quadratic *X*_2_, resulting in a positive effect on resveratrol extraction. Variables *X*_1_, *X*_3_, *X*_1_*X*_2_, *X*_2_*X*_3_, significantly impacted the resveratrol content, showing an excellent coefficient of correlation of the predicted model (R^2^ = 0.9850). The extraction yield indicated a linear effect for *X*_1_ and *X*_3_ and a quadratic impact for *X*_2_, an R^2^ = 0.8993. Data analysis of the extraction yield showed that the mathematical model could predict the effect of the interactions for variables *X*_1_, *X*_2_, and *X*_3_ on UAE from *P. cuspidatum*.

### 2.2. Response Surface Plot Analysis

**Figure 1:**
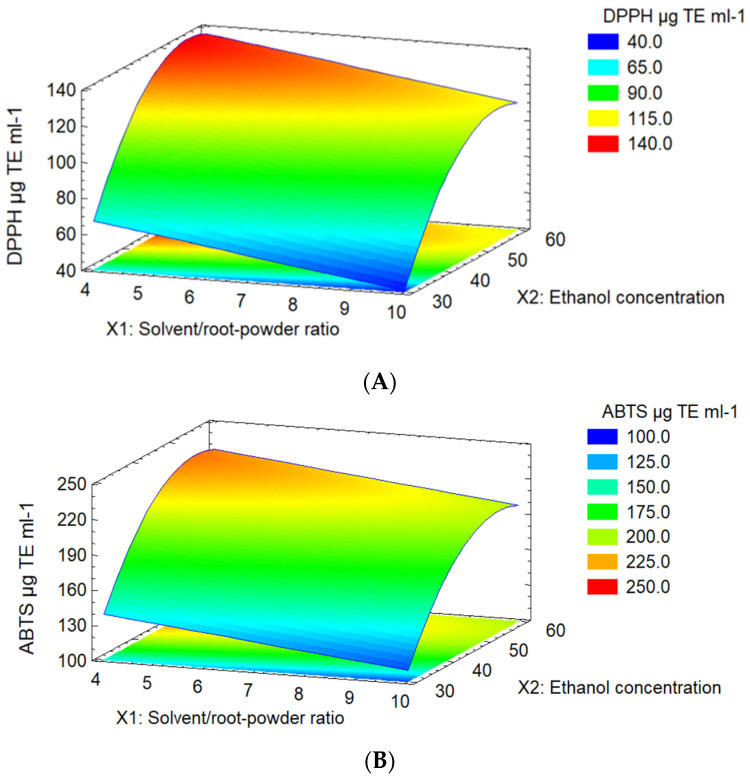
Surface plot of the combined effect of the independent variables on antioxidant activities: (**A**) DPPH (*X*_1_*X*_2_) and (**B**) ABTS^+^ radical (*X*_1_*X*_2_). *X*_1_ = solvent/root-powder ratio and *X*_2_ = ethanol concentration.

**Figure 2:**
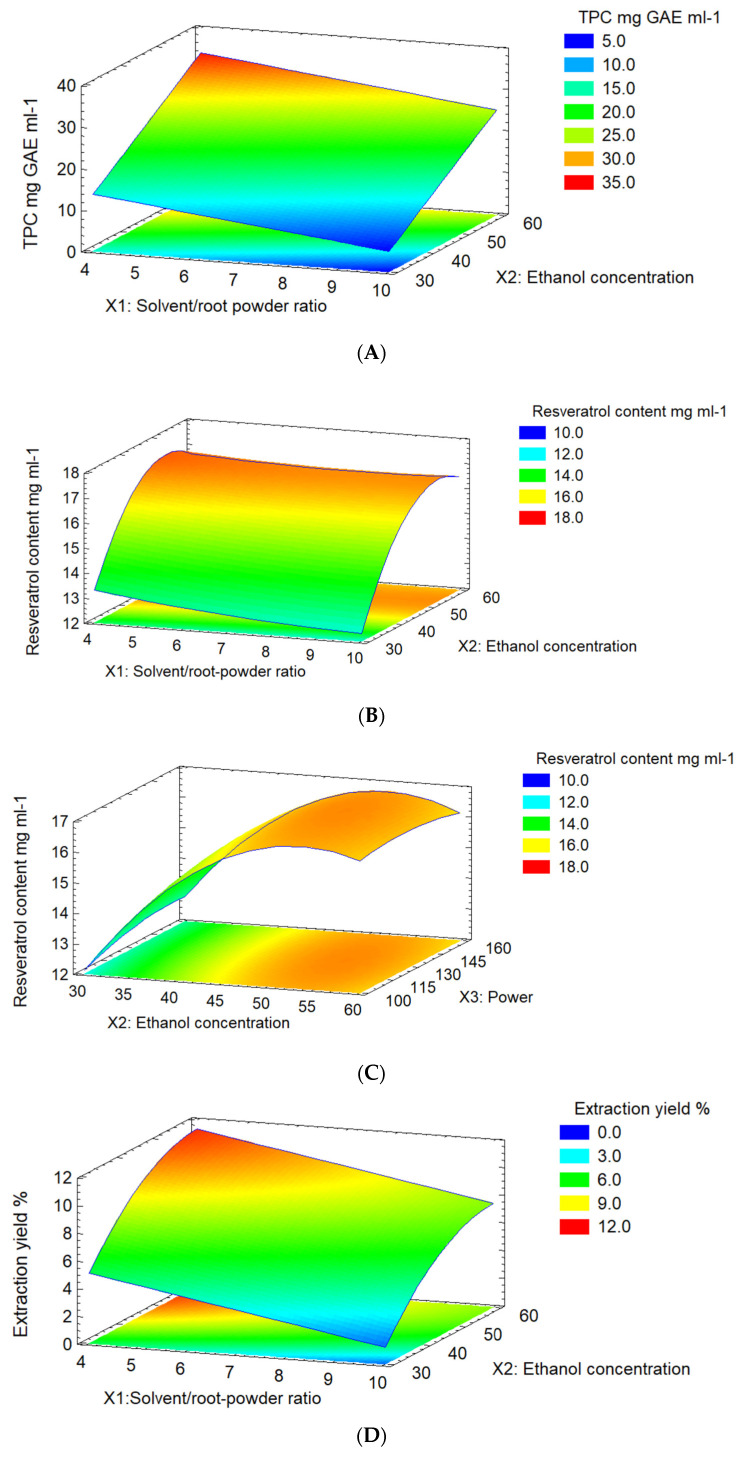
3D plots for interaction variables for (**A**) TPC (*X*_1_*X*_2_), (**B**) resveratrol content (*X*_1_*X*_2_) y (**C**) resveratrol content (*X*_2_*X*_3_), and (**D**) extraction yield (*X*_2_*X*_3_). *X*_1_ = solvent/root-powder ratio, *X*_2_ = ethanol concentration, and *X*_3_ = ultrasonic power.

The 3D response surface plots show the impacts of independent variables on the antioxidant capacity, TPC, resveratrol content, and extraction yield of *P. cuspidatum* (Figure 1 and Figure 2). Results revealed high antioxidant activities with increasing ethanol concentration (from 45% to 60%) and decreasing solvent/root-powder ratio (from 4 mL g^−1^ to 7 mL g^−1^), reaching maximum values of 135.1 µg TE mL^−1^ (IC_50_ = 78 µg TE mL^−1^) and 230.4 µg TE mL^−1^ (IC_50_ = 158 µg TE mL^−1^) for DPPH and ABTS+, respectively.

TPC showed a maximum concentration of 33.2 mg GAE mL^−1^ at higher ethanol concentrations (45% to 60%) and low solvent/root-powder ratios (4 mL g^−1^ to 7 mL g^−1^) (Figure 2A).

Resveratrol content (Figure 2B,C) showed a highly significant impact (*p* < 0.001), with resveratrol content increasing to 16.7 mg mL^−1^ when the ethanol concentration was higher than 50%. These results suggest that solvent/root-powder ratio, ultrasonic power, and interactions impacted the resveratrol concentration. The maximum extraction yield (Figure 2D), corresponding to 12.43%, reached a lower solvent/root-powder ratio (4 mL g^−1^ to 7 mL g^−1^) and high ethanol concentration (45 to 60%).

The regression coefficients to calculate the predicted response for the antioxidant activities of ABTS+ and DPPH, TPC, resveratrol content, and extraction yield were performed using a reduced second-order polynomial equation (Table 3).

**Figure 3:**
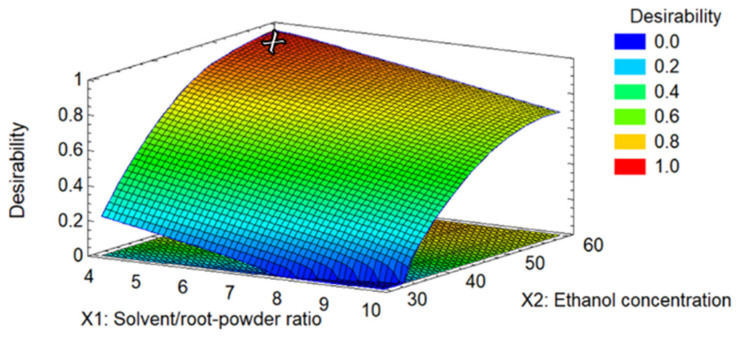
Desirability 3D plot; the cross mark represents the desirability for optimal extraction conditions.

The optimal predicted value was obtained in experimental run 14, corresponding to a solvent/root-powder ratio of 4, 60% ethanol concentration, and 128.5 W of ultrasonic power, corresponding to 58 KJ/g. The predicted values for the antioxidant activity of DPPH and ABTS+ corresponded to 136.625 µg TE mL^−1^ and 227.575 µg TE mL^−1^, respectively. For TPC, the expected value was 34.068 mg GAE mL^−1,^ and resveratrol content and extraction yield were 15.56 mg mL^−1^ and 11.36%, respectively. The predicted desirability value for optimal extraction of *P. cuspidatum* was 0.9661 (Figure 3). The experimental conditions were validated using an independent experiment, finding 136.2 µg TE mL^−1^, 195.4 µg TE mL^−1^, 29.95 mg GAE mL^−1^, 16.72 mg mL^−1^, and 12.3% for DPPH, ABTS+, TPC, resveratrol, and extraction yield, respectively.

Analysis of the infusion of *P. cuspidatum* root powder indicates that the values for the antioxidant activities of DPPH and ABTS+ were 103.75 µg TE mL^−1^ and 147.78 µg TE mL^−1^, respectively. In addition, the TPC value was 0.4024 mg GAE mL^−1^. The resveratrol content and extraction yield were 0.044 mg mL^−1^ and 0.340%, respectively.

### 2.3. Cytotoxic Assay and EC_50_ Value

**Figure 4:**
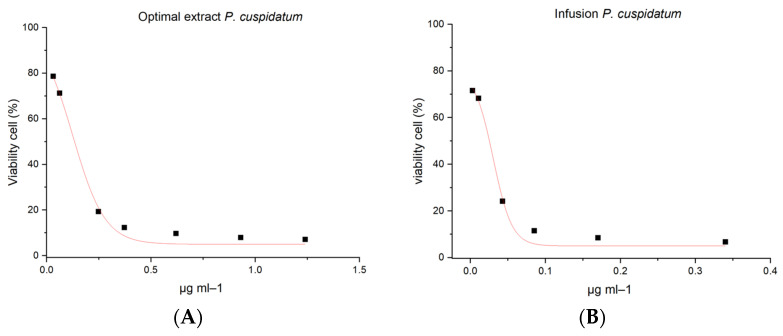
Effect of *P. cuspidatum* extracts on viability of cells at different concentrations for 24 h. (**A**) Caco-2 cells treated with diluted optimized extract, (**B**) Caco-2 cells treated with direct infusion. Data show mean ± SEM from three independent experiments.

**Figure 5:**
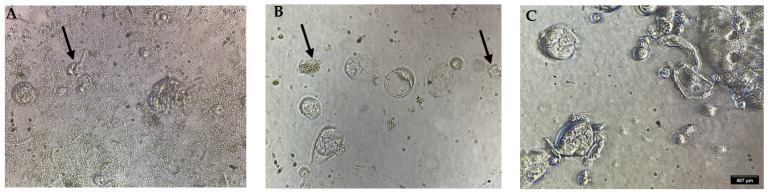
Microphotography of Caco-2 cells treated with *P. cuspidatum* extracts after 24 h: (**A**) small cells after the treatment with optimized extract; (**B**) apoptotic cells induced by aqueous infusion and (**C**) non-treated cells (negative control). Arrows indicate the cells at different treatments.

The optimized extract was diluted (1:110) in MEM (Minimal Eagle’s Medium), and the infusion was used to directly compare the cytotoxicity on colorectal cancer cells for 24 h. The results obtained for the optimized extract demonstrate that the viability of Caco-2 cells decreases at low concentrations in a dose-depending manner (1.24 µg mL^−1^ to 0.03 µg mL^−1^), and the EC_50_ value corresponded to 0.125 ± 0.008 (R^2^ = 0.9913) (Figure 4A). In contrast, the infusion extraction of *P. cuspidatum* showed low viability with an estimated EC_50_ of 0.03 ± 0.002 µg mL^−1^ (R^2^ = 0.9892) (Figure 4B). Figure 5A shows that Caco-2 cells treated with optimized extraction were smaller and had low cell confluency compared to untreated cells (Figure 5C). Cells treated with the infusion of *P. cuspidatum* show higher confluency and apoptotic bodies, characteristic of the induction of apoptotic cell death (Figure 5B), compared to those treated with the optimized extract (Figure 5C).

### 2.4. Compounds Identified by UPLC-Mass Spectrometry (MS)

**Figure 6:**
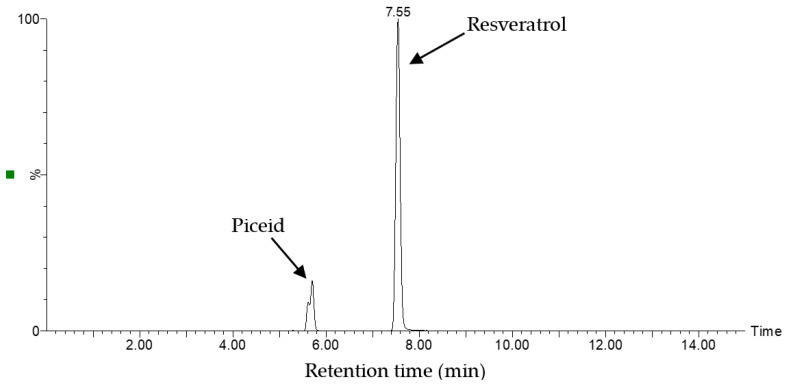
Chromatogram of the optimized extract from *P. cuspidatum* obtained by mass spectrometry (MS).

Figure 6 shows the chromatogram of the optimized extract from *P. cuspidatum* obtained through MS analysis. The spectra revealed two main peaks attributed to stilbene compounds: piceid (RT = 5.71) and resveratrol (RT = 7.55). Thus, these results indicate that piceid and resveratrol are the major bioactive components in the optimized extract.

## 3. Discussion

Bioactive compounds have medicinal benefits and are extracted from fresh or dried plants using different extraction methods and solvents [26]. Solvents impact the recovery and quality of bioactive compounds, but can degrade them at high temperatures [27]. Water is used to prepare beverages with an affinity for hydrophilic molecules, such as phenolic compounds, proteins, and carbohydrates. Studies have reported that the low dielectric constant and low polarity of hot water increase the diffusion of compounds, improving the extraction of lipophilic bioactive compounds. On the other hand, ethanol is used to extract bioactive compounds, such as polyphenols, flavonoids, alkaloids, and terpenes [28,29]. Several factors, such as plant type, ethanol concentration, and extraction time, impact the extraction yield. The ethanol–water concentration increases the polarity of the solvent, improving the efficiency of the extraction of nonpolar phytochemicals [30].

Usually, resveratrol is extracted with 95% ethanol from the root of *P. cuspidatum* using a refluxing method followed by liquid–liquid extraction with organic solvents [25]. Kuo et al. [18] found that when applying a temperature of 70 °C, 60% ethanol concentration, and 120 W ultrasonic power, the amount of resveratrol was 3.9 mg/g. Jia et al. [31] reported high flavonoid concentration (94.5%) using UAE- and CCRD-extracted flavonoids from *P. cuspidatum* using an ethanol concentration of 60%, solid–liquid ratio of 1:20 g mL^−1^, extraction temperature of 45 °C, extraction time of 34 min, and ultrasonic power of 80 W. Ruan et al. [32] reported an extraction yield value for resveratrol and emodin of 2.564 ± 0.121 mg mL^−1^ and 2.804 ± 0.108 mg mL^−1^, respectively, and a high scavenging capacity. Our results showed that resveratrol content at the optimized extract conditions was higher than those reported in the literature.

The standard methods to evaluate the antioxidant capacity of foods employ the stable radicals DPPH and the cation ABTS+. Results obtained from antioxidant assays reported the necessary antioxidant concentrations to reduce radicals [33]. Becze et al. [34] obtained extracts with a high antioxidant capacity of 34.623 µg AAE mL^−1^ and 182.4 µL of resveratrol content from *F. regala* leaves. UAE of *Arachis repens*, known as peanut ass, showed high concentrations of trans-resveratrol and total polyphenolics, demonstrating a high level of DPPH free radical inhibition (70%). Polyphenolic compounds can reduce the Folin–Ciocalteu reagent under alkaline conditions, which yields a colored product [35]. El Moussaoui et al. [36] evaluated the antioxidant activity and total polyphenols of extract from *Whitania frutescens* L., and found that roots are 67 times richer in polyphenols (53.3 ± 1.2 mg GAE/g).

The optimized ethanolic extract showed higher cytotoxic activity than the infusion extract cont due to the higher resveratrol concentration found in the ethanolic extract. Similar results were found by Youmbi and coworkers [37]. They found that crude extract obtained from the leaves of *Brucea antidysenterica* induces cell death mediated by caspases on drug-resistant cancer cells, such as CCRF-CEM, a human leukemia cell line, at a low concentration (from 12.42 µg mL^−1^ to 38.70 µg mL^−1^). Other authors reported that ethanol extracts of *P. cuspidatum* demonstrated efficacy on the loss of viability of cells in oral, breast, and ovarian cancer at lower concentrations, EC_50_: <50 µg mL^−1^ [17,38,39].

On the other hand, tea or infusion is widely used for its beneficial properties and low cost. Traditionally, *P. cuspidatum* is commonly used as part of traditional Chinese medicine, and is prepared as a herbal infusion in boiling water for a few minutes. Kosovic et al. [40] evaluated the stability of resveratrol from *Vitis vinifera* L. at high temperatures, finding a better release of stilbenes such as trans-resveratrol. However, if a prolonged time was applied, the polyphenolic compounds could be degraded. The effect of green tea on rat hepatocyte cells was evaluated by Schmidt et al. [41], and the results show that cell viability decreased at 500 µg mL^−1^ of the extract. Aqueous extract of *Reynoutris japonica* Houtt root, a Polygonaceae family, was not cytotoxic in SK-Hep1 and Huh7 cell lines, but *in vitro* results indicated that at a concentration of 20 µg mL^−1^ inhibited wound recovery and invasion in Huh7 cells [42].

The main compounds in *P. cuspidatum* root extracts have been investigated using chromatographical analysis. Yi et al. [43] reported that the *P. cuspidatum* rhizome is rich in stilbenes (piceid and resveratrol) and anthraquinones (emodin-8-*O*-*β*-*_D_*-glucoside, and emodin). These compounds are commonly used as an indicator of quality assessment for herbal extracts. Vastano et al. [44] evaluated two varieties of *P. cuspidatum* root extracts (Hu Zhang and Mexican Bamboo). They identified three stilbene glucosides: piceatannol glucoside, resveratroloside, and piceid. By comparison, our results showed that the optimized extract of *P. cuspidatum* root contains piceid and resveratrol as the major bioactive compounds.

## 4. Materials and Methods

### 4.1. Plant Material and Reagents

Dried root powder of *P. cuspidatum* with particle size 80 mesh (~0.177 mm) was obtained from Herbal Mexico (Tlalnepantla de Baz, Mexico). Pure ethyl alcohol, 6-hydroxy-2,5,7,8-tetramethyl chroman-2-carboxylic acid (Trolox), 2,2-diphenyl-1-picrylhydrazyl (DPPH), 2,2′-Azino-bis(3-ethylbenzothiazoline-6-sulfonic acid) diammonium salt (ABTS+), sodium persulfate, Folin–Ciocualteu phenol reagent, and resveratrol standard (purity > 99%) were purchased from Sigma-Aldrich (St. Louis, MO, USA). Gallic acid and sodium carbonate (Na_2_CO_3_) were obtained from Jalmek (San Nicolás de Los Garza, NL, Mexico). MEM and Fetal Bovine Serum (FBS) were purchased from Biowest, and Tripsine-EDTA solution and Resazurin sodium salt from Sigma-Aldrich.

### 4.2. Optimization of Ultrasonic-Assisted Extraction (UAE)

*P. cuspidatum* powder was dispersed in ethanol, according to Table 1. Then, dispersions were sonicated using an Elmasonic p70H ultrasonic bath (37 kHz, Elma Schmidbauer GmbH, Singen, Germany) following the conditions of the Box–Behnken design. After applying the ultrasound, the samples were centrifuged at 4000 rpm for 15 min. The supernatant was collected and stored at −20 °C until analysis. As a control, an infusion of 10 g of root powder was dispersed into 200 mL of boiling water; afterward, the solution was filtrated to remove sediment and stored at −20 °C until analysis.

### 4.3. Experiment Design Strategy and Statistical Analysis

RSM optimized the UAE extraction parameters for DPPH, ABTS+, TPC, resveratrol concentration, and extraction yield from *P. cuspidatum*. In a three-level, three-factorial Box–Behnken design, including five replicates of the central point, 17 runs were analyzed in random order (Table 4). The independent variables were solvent/root-powder ratio (*X*_1_, mL g^−1^), ethanol concentration (*X*_2_, %), and ultrasonic power (*X*_3_, W). Temperature and extraction time were fixed at 45 °C and 30 min, respectively.

RSM was applied to obtain the optimal UAE conditions for each raw material. A second-order polynomial equation derived from the RSM was used to calculate the predicted response (Equation (1)):

(1) $$Y=\beta_{0}+\overset{k}{\underset{i=1}{\sum }}\beta_{i}X_{i}+\overset{k}{\underset{i=1}{\sum }}\beta_{ii}X_{ii}+\overset{k}{\underset{i>j}{\sum }}\beta_{ij}X_{j}+E$$

*Y* is the response variable, *X_i_* is the coded or uncoded value for the factors evaluated, *β*_0_ is a constant, *β_i_* is the main effect of the coefficient for each variable, *β_ij_* represents the interaction effect coefficients, and *E* is the error of the model. ANOVA evaluated significant interactions of the model (*p* < 0.05). The coefficient of determination was quantified (R^2^ and adjusted R^2^). The predicted values for antioxidant activity (DPPH and ABTS+), TPC, resveratrol content, and extraction yield were maximized to establish the optimal UAE via RSM. An additional extraction was conducted using the optimal predicted conditions to verify the model’s suitability. Applied energy was estimated according to Strieder et al. [45]: energy = [ultrasonic power × time extraction]/mass of the sample. The regression coefficients were used to generate 3D surface plots from the fitted polynomial equation. Also, the regression coefficients were used to visualize the relationship between the response variable and experimental levels and predict the optimum conditions.

### 4.4. Determination of Extraction Yield

The extraction yield (%) was quantified by vaporization utilizing 2 mL of crude extract in an oven at 40 °C until the dry mass was obtained. The results were shown as a mass of total extractable solids per 100 g of dry plant material (%).

### 4.5. Resveratrol Quantification Using Spectrophotometry UV–Vis

UV–Vis spectroscopy was used to assess the concentration of resveratrol in the crude extracts. Samples were compared with a standard curve of resveratrol in ethyl alcohol. Ethyl alcohol was utilized as a blank for background correction. Absorbance was read at 312 nm using a multiwell plate reader (TECAN infinite pro-200, Trading AG, Steinhausen, Switzerland).

### 4.6. In Vitro Antioxidant Capacity

#### 4.6.1. DPPH Radical Scavenging Assay

For the DPPH stable radical scavenging assay, samples (ethanolic samples 1:400, infusion 1:2), blank (ethyl alcohol), or Trolox standard were added to a 96-well microplate. Subsequently, DPPH solution was added to each well and allowed to stand for 30 min in the dark before reading absorbance at 517 nm. All experiments were realized in triplicate. The capacity to scavenge the DPPH radical was presented as µg of Trolox equivalent (TE) mL^−1^ [46].

#### 4.6.2. ABTS+ Assay

For the ABTS+ test, in a 96-well microplate the sample (ethanolic samples 1:400, infusion 1:2), blank (ethyl alcohol pure), or Trolox standard were added. Subsequently, the ABTS+ working solution was added to each well and allowed to stand for 5 min in the dark before reading absorbance at 734 nm [22]. All experiments were realized in triplicate. The results for antioxidant activity in the ABTS+ radical test were presented as µg of Trolox equivalent (TE) mL^−1^ [46].

#### 4.6.3. Total Polyphenolic Compounds

Polyphenols were measured using Folin–Ciocalteu’s method. The samples (ethanolic samples 1:400, infusion 1:2) and blank or gallic standard curves were placed in a 96-well plate. Next, 0.5 N Folin–Ciocalteu phenol reagent and 75 µL Na_2_CO_3_ solution (0.1 mg mL^−1^) were added. Absorbance was measured at 765 nm and the results expressed as mg GAE per mL [47].

### 4.7. Caco-2 Cell Culture and Cytotoxic Assay

The cytotoxic assay for the optimal extraction of *P. cuspidatum* was evaluated using a resazurin reduction assay [48]. The optimal extract was diluted with MEM to obtain different concentrations. The Caco-2 cell line (HTB-37 ATCC), a human colorectal adenocarcinoma, was cultured in MEM supplemented with 10% FBS and 1% penicillin/streptomycin. Briefly, cells were incubated until reaching ~80% confluence and seeded in a 96-well plate at 5 × 10^3^ cells per well at 37 °C and 5% CO_2_. After 24 h, MEM was replaced with 100 μL of the extracts at different concentrations and incubated for 24 h (37 °C and 5% CO_2_). After that, 20 μL of resazurin solution (0.2 mg mL^−1^) was added and incubated for 3 h at 37 °C. The fluorescence emitted by resorufin, metabolized from resazurin by viable cells, was measured (excitation 560 nm, emission 590 nm). A negative control (MEM medium) and positive control (DMSO 10%) were used. Percentage viability and effective concentration 50 (EC_50_) of crude extracts and the infusion of *P. cuspidatum* were calculated using OriginPro 2018 Software (Origin Lab Corporation, Northampton, MA, USA). The cell viability was calculated as shown:

% cell viability = *I*_s_/*I*_c_ × 100%

where: *I_s_* is the absorbance of the cells exposed to the extracts and *I_c_* is the absorbance of the cells without exposure to the extracts

### 4.8. Chromatography Analysis by Mass Spectrometry (MS/MS)

Mass spectrometry (MS) analysis was performed using a Waters Xevo TQ-S instrument (Waters, Milford, MA, USA). The conditions used for identifying the bioactive compounds from the optimized extract by comparing fingerprint and MS data were those previously reported [49,50]. Resveratrol and piceid were identified using multiple reaction monitoring (MRM), a sensitive method of targeted MS. For acquisition and data processing, MassLynx V4.1 software was employed (Waters, Milford, MA, USA).

### 4.9. Statistical Analysis

The results obtained were analyzed using ANOVA and Tukey’s means comparison analysis. Data from the Box–Behnken experimental design were examined using the least square multiple regression methodology to fit the polynomial models for UAE optimization. Data analysis and response surfaces were conducted using Statgraphics Centurion XVI. I software (Statistical Graphics Corp., Manugistics, Inc., Cambridge, MA, USA). Significance was established at *p* ≤ 0.05. All experiments were conducted in triplicate.

## 5. Conclusions

Ultrasonic-assisted extraction from *P. cuspidatum* roots was effectively developed. The extraction efficiency, antioxidant activity, resveratrol content, and total polyphenolic compounds could be further improved through BBD experimental design and response surface methodology. A second-order model was obtained to describe the relationship between the responses and their interactions with independent variables *X*_1_ (solvent/root powder ratio), *X*_2_ (ethanol concentration), and *X*_3_ (ultrasonic power at 45 °C for 30 min). The evaluated parameters affected antioxidant activity (DPPH, ABTS+), total polyphenolic compounds, resveratrol content, and extraction yields from the crude extract from *P. cuspidatum* root. The optimized extract showed higher cytotoxic activity than the traditional infusion of *P. cuspidatum* root against Caco-2 colorectal cancer cells. Due to the complex mixture in the optimized extract of *P. cuspidatum*, future research is required to identify and quantify all the bioactive compounds and to determine the mechanism by which this extract induces cell death.

## Figures and Tables

**Table 1:** Experimental values of antioxidant activity, total polyphenolic compounds, resveratrol concentration, and extraction yield obtained by UAE with a Box–Behnken design.

Experimental Run	Independent Variables			Responses				
				Antioxidant Capacity (µg TE mL^−1^)		TPC	Resveratrol	Extraction Yield
	*X*_1_ (mL g^−1^)	*X*_2_ (%)	*X*_3_ (W)	DPPH	ABTS+	(mg GAE mL^−1^)	Concentration (mg mL^−1^)	(%)
1	7	45	129	103.5	190.3	18.9	16.3	6.6
2	7	45	129	116.2	198.4	18.9	16.5	8.5
3	7	45	129	126.7	214.7	27.9	16.3	9.3
4	7	45	129	109.0	194.3	19.4	16.4	7.5
5	7	45	129	109.7	195.3	19.1	16.5	7.3
6	4	45	107	132.9	222.2	25.0	16.5	9.9
7	7	30	150	52.5	126.6	8.0	12.7	3.6
8	10	45	150	103.5	188.2	16.8	16.3	5.6
9	10	60	107	103.3	190.4	19.3	16.2	6.6
10	7	30	129	51.1	121.2	6.8	12.0	2.7
11	7	60	150	126.7	215.8	29.3	16.6	9.4
12	4	45	107	133.8	229.0	27.6	16.7	10.1
13	10	30	150	51.3	119.5	5.8	12.4	2.9
14	4	60	107	135.1	230.4	33.2	16.6	12.4
15	4	30	129	60.3	129.4	8.8	13.6	3.3
16	7	60	129	129.4	215.8	29.1	16.5	9.3
17	10	45	129	99.9	197.1	17.7	16.2	5.24

**Table 2:** ANOVA results of the correlated polynomial model for antioxidant activity, TPC, resveratrol content, and extraction yield.

Response	Source	Sum of Squares	df	Mean Square	*F*-Value	*p*-Value
ABTS+	*X*_1_	1676.21	1	1676.21	18.75	0.0123 *
	*X*_2_	15,815.3	1	15,815.3	176.94	0.0002 **
	*X*_3_	55.6513	1	55.6513	0.62	0.4742
	*X*_2_ *^2^*	5082.14	1	5082.14	56.86	0.0017 *
	Lack of fit	555.787	8	69.4734	0.78	0.6481
	Pure error	357.52	4	89.38		
	Cor Total	23,542.6	16			
	*R*_2_ = 0.9612					
	*R*_2*adju*_ = 0.9483					
DPPH	*X*_1_	1352.0	1	1352.0	17.16	0.0143 *
	*X*_2_	9751.06	1	9751.06	123.80	0.0004 **
	*X*_3_	1.90125	1	1.90125	0.02	0.8841
	*X*_2_^2^	2934.15	1	2934.15	37.25	0.0036 *
	Lack of fit	341.858	8	42.7323	0.54	0.7860
	Pure error	315.068	4	78.767		
	Cor Total	14,696.0	16			
	*R*^2^ = 0.9552					
	*R*^2^*_adju_ =* 0.9403					
TPC	*X*_1_	153.125	1	153.125	9.80	0.0351
	*X*_2_	830.281	1	830.281	53.16	0.0019 *
	*X*_3_	3.00125	1	3.00125	0.19	0.6838
	Lack of fit	100.61	9	11.1789	0.72	0.6901
	Pure error	62.472	4	15.618		
	Cor Total	1149.49	16			
	*R*^2^ = 0.8581					
	*R*^2^*_adju_ =* 0.8253					
Resveratrol content	*X*_1_	0.66125	1	0.66125	66.12	0.0012 *
	*X*_2_	28.88	1	28.88	2888.00	0.0000 **
	*X*_3_	0.10125	1	0.10125	10.12	0.0335 *
	*X*_1_ *X*_2_	0.16	1	0.16	16.00	0.0161 *
	*X*_2_ *X*_3_	0.09	1	0.09	9.0	0.0399 *
	*X*_1_^2^	0.0796053	1	0.0796053	7.96	0.0478
	*X*_2_^2^	14.2164	1	14.2164	1421.64	0.0000 **
	*X*_3_^2^	0.0532895	1	0.0532895	5.33	0.0822
	Lack of fit	0.505	4	0.12625	12.62	0.0154 *
	Pure error	0.04	4	0.01		
	Cor Total	44.8424	16			
	*R*^2^ = 0.9878					
	*R*^2^*_adju_* = 0.9756					
Extraction yield	*X*_1_	29.6835	1	29.6835	26.46	0.0068 *
	*X*_2_	79.317	1	79.317	70.72	0.0011 *
	*X*_3_	0.09245	1	0.09245	0.08	0.7883
	*X*_2_^2^	9.45017	1	9.45017	8.43	0.0440 *
	Lack of fit	8.78113	8	1.09764	0.98	0.5486
	Pure error	4.48648	4	1.12162		
	Cor Total	131.811	16			
	*R*^2^ = 0.8993	29.6835				
	*R*^2^*_adju_* = 0.8658					

* Statistical significative (*p* < 0.05), ** high statistical significative (*p* < 0.001).

**Table 3:** Reduced polynomial equations for antioxidant capacity, TPC, resveratrol content, and extraction yield.

Response	Reduced Equations
ABTS^+^	ABTS+ = −223.86 − 4.82*X*_1_ + 16.82*X*_2_ + 0.12*X*_3_ − 0.15*X*_2_^2^
DPPH	DPPH = −193.35 − 4.33*X*_1_ + 12.86*X*_2_ − 0.02*X*_3_ − 0.12*X*_2_^2^
TPC	TPC = −4.51 − 1.46*X*_1_ + 0.68*X*_2_ + 0.03*X*_3_
Resveratrol content	Resveratrol content = −10.40 − 0.51*X*_1_ + 0.89*X*_2_ + 0.088*X*_3_ + 0.015*X*_1_^2^ + 4.4 × 10^−3^*X*_1_ *X*_2_ − 8 × 10^−3^*X*_2_^2^ − 4.6 × 10^−4^ *X*_2_ *X*_3_ − 2.4 × 10^−4^ *X*_3_^2^
Extraction yield	Extraction yield = −11.26 − 0.64*X*_1_ + 0.81*X*_2_ + 0.017*X*_3_ − 6.64 × 10^−3^*X*_2_^2^

*X*_1_ = solvent/root-powder ratio, *X*_2_ = ethanol concentration, *X*_3_ = ultrasonic power.

**Table 4:** Levels of variables for experimental design.

Independent Variable	Coded Levels		
	−1	0	1
*X*_1_	4	7	10
*X*_2_	30	45	60
*X*_3_	107	128.5	150

## Data Availability

Data will be made available on request.
